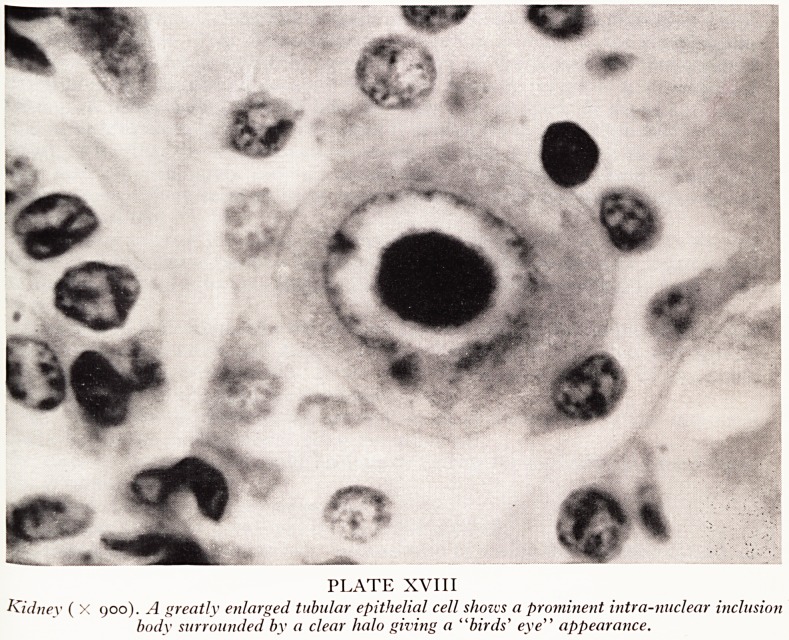# Cytomegalic Inclusion-Body Disease

**Published:** 1965-07

**Authors:** O. C. Lloyd


### CYTOMEGALIC INCLUSION-BODY DISEASE

*A Clinico-Pathological Conference held in the University of Bristol on 9th March, 1965*

CHAIRMAN: DR. O. C. LLOYD

*Dr. Beryl Corner:* This female child was the first of a pair of twins born at Mortimer House Hospital. The mother was an unmarried girl aged 24. It was her first pregnancy and was supposed to have been of 36 weeks duration. This child, born by normal vertex delivery, weighed only 2 lb. 9 oz. and so was immediately transferred to the premature baby unit at Southmead Hospital where she arrived $1\frac{1}{4}$ hours after birth. The whole of her body was found to be covered by petechial haemorrhages which in places merged to form larger lesions resembling bruises. She had a very large spleen and a very large liver and was thought to be very slightly jaundiced. It was soon realized that this baby had some quite unusual problem and our first thoughts were that either she was suffering from some overwhelming infection, which seemed most likely, or that she had some blood disorder. The mother's blood group was AB, Rh-positive so haemolytic disease seemed very unlikely unless a very unusual type of incompatibility was present.

Investigations were started at once. The conjugated serum bilirubin was raised to 4 mg per cent. The unconjugated bilirubin (2·5 mg per cent) was within the normal range for a child of this age. The haemoglobin was slightly reduced (90 per cent) and there was a marked thrombocytopenia (62,000 per cu. mm). White blood cells were not abnormal and there were 70 nucleated red cells per 100 white cells; this is rather more than one would expect although some nucleated red cells are normally present on the first day of life. Wassermann reaction and toxoplasmosis tests were negative and no cytomegalic inclusion bodies were found in several specimens of urine; the urine was also normal on routine examination. The serum alkaline phosphatase was only 17 units at this time but rose to 53 units by the time the child was a fortnight old. The S.G.P.T. was 113 units at first and later rose to 320 units. The Coombs test was negative. Stools were pale. Prothrombin index was 55 per cent.

It now appeared that the child had some form of hepatitis and in view of this and the low platelet count, steroid therapy was commenced but seemed to have little effect.

When the child was about a month old, Mr. A. G. McPherson carried out a liver biopsy. This revealed an active hepatitis with widespread infiltration of the liver tissue by round cells and polymorphs with patchy liver cell degeneration and some foci of haemopoiesis; the bile ducts were normal and no inclusion bodies or giant cells were seen. In spite of antibiotic therapy and a blood transfusion the child's condition continued to deteriorate but rather surprisingly she survived for six weeks. Two days before death pus cells were found in the urine and she seemed to have a terminal urinary infection. She became lethargic, skin petechiae were very numerous again and she died.

The eyes were examined on several occasions by Mr. C. A. Brown. No abnormalities were found until one day before she died when the cornea appeared hazy and there was thought to be some choroiditis in both fundi. The other twin appeared perfectly normal at birth and was followed up for a time, and when we knew the diagnosis of her sister we did investigate her for the same condition with negative results. I am afraid, however, that the mother has now ceased to attend the out-patient clinic.

*Dr. R. Martin:* Was the liver biopsy a needle biopsy or an open biopsy?

*Dr. Corner:* An open biopsy.

*Dr. Martin:* Were the extra-hepatic bile ducts examined?

*Dr. Corner:* I am sure Mr. McPherson would have examined them.

*Dr. N. J. Brown:* Yes, he did look at them and they were normal.

*Dr. Martin:* Were the twins identical?

*Dr. Corner:* They were of different blood groups and so I don't think they can have been.

*Dr. D. W. Barritt:* The diagnosis of hepatitis would account for the jaundice, the pale stools and the low prothrombin index, but how did you account for the low platelet count?

*Dr. Corner:* We haven't accounted for that, it is true.

*Dr. Barritt:* Can you tell us something about neonatal hepatitis?

*Dr. Corner:* We see more cases of neonatal hepatitis than we do of congenital anomalies of the biliary tract. It can present as in this case at birth with jaundice, petechiae and bruising, or it may develop during the first 4 weeks of life. Whenever we get one of these cases we always carry out full investigations but if it survives we very rarely find any specific cause for the hepatitis. Some of them are probably due to viruses, and toxoplasmosis accounts for others, but in most of them the aetiology is unknown. Cytomegalic inclusion-body disease is always considered, but neither in this baby nor in any of the others we have investigated have we found any of the typical cells in the urine.

*Dr. Lloyd:* Do these cases usually present with purpura?

*Dr. Corner:* The severe ones do.

*Dr. Barritt:* Can anybody think of a disease where hepatitis is accompanied by low platelet counts?

*Prof. A. V. Neale:* We have to consider of course whether the reduction of platelets is the cause of the purpura or the result of the purpura.

*Dr. Lloyd:* I think rather than speculate further at this stage it might be a good idea for Dr. Brown now to tell us what the disease really was and demonstrate its morbid anatomy. We can then start fitting our jig-saw puzzle together.

*Dr. Bennett:* Could I just ask if the child was febrile at any stage.?

*Dr. Corner:* No, not really. The highest temperature was 99·4°F on the day of death.

*Dr. Brown:* (presenting the necropsy findings). This disease is something of a rarity but I make no apology for bringing it up since I find that a recent edition of a well-known textbook of pathology for undergraduates has quite a little passage on it!

The body was deeply jaundiced, and emaciated; it actually weighed slightly less than it did at birth. It had a large dark green liver (103 g) which was rather tough and fibrous in texture; the bile ducts were normal. The spleen was enlarged (16 g) and rather pale. The kidneys were pale green in colour but otherwise normal in appearance and the other organs showed no macroscopic abnormalities.

The diagnosis was revealed by histological examination. The typical changes are best seen in the kidney (Plate XVII). The glomeruli and many of the tubules are normal but other tubules show enormously enlarged lining cells containing striking intra-nuclear and intracytoplasmic inclusion bodies. The former are centrally placed within the nucleus and surrounded by a clear halo giving a "bird's eye" appearance (Plate XVIII). The intracytoplasmic inclusions are smaller and multiple, occurring either in groups in one part of the cell or scattered throughout the whole of the cell body.

The liver shows a severe and active hepatitis as in the biopsy with round cell and polymorphonuclear infiltration. There is now some fibrosis in the portal tracts which was not present in the earlier specimen and the bile duct epithelium now shows occasional enlarged cells containing inclusion bodies.

Similar cytomegalic appearances with inclusion bodies were found in the salivary gland ducts, in the pancreas, and in small numbers in the lungs.

This then is a case of cytomegalic inclusion-body disease. It is a condition which

PLATE XVII

*Kidney* (× 120). *Many of the renal tubular epithelial cells are greatly enlarged and contain intra-nuclear and intra-cytoplasmic inclusion bodies.*

PLATE XVIII

*Kidney* (× 900). *A greatly enlarged tubular epithelial cell shows a prominent intra-nuclear inclusion body surrounded by a clear halo giving a "birds' eye" appearance.*

affects infants and young children, often presents as hepatitis and exhibits these striking pathological appearances in cells of epithelial type throughout the body. It is supposed to be caused by a virus which appears to be related to the mouse salivary gland virus which causes similar changes in the salivary glands of mice. Some people have claimed that if you examine routinely the salivary glands of children at necropsy you will find inclusion bodies in a proportion of cases which have no clinical evidence of the disease. This has not been our experience at Southmead Hospital where for many years we have routinely examined salivary glands without finding a single case.

*Dr. Lloyd:* So the fact that you were not able to find the typical changes in the liver biopsy was just bad luck.

*Dr. Brown:* Yes. Needless to say we have re-examined the biopsy slides but we cannot see any.

*Dr. Martin:* Did you examine the post-mortem urinary deposit?

*Dr. Brown:* No.

*Dr. Lloyd:* When you find affected cells in the urinary deposit are they cells derived from the epithelium of the bladder?

*Dr. Brown:* Presumably.

*Dr. Lloyd:* Did you find them in the bladder epithelial cells in the necropsy material?

*Dr. Brown:* I did not look (subsequent examination reveals that they are in fact present). They could equally well be derived from renal tubular epithelium.

*Dr. Lloyd:* Yes they could but then you would expect them to be associated with granular casts. Would Dr. Cayton please tell us something about the organism responsible for these cases.

*Dr. H. R. Cayton:* Speaking rather "off the cuff" I would say that the virus of this disease is somewhat similar to the herpes virus in the sense that it is a virus which is apparently very widely distributed in the population but only produces acute clinical signs in a small number. I say this because antibody to this virus is present in a high proportion of adults. This is neutralizing antibody in contrast to the complement-fixing antibody which is found in the acute stage of the disease and for a period afterwards in the discovered cases. I think this must be regarded as a congenital infection. It is fascinating to think how the other twin escaped. If she did escape it would be very interesting to get some serum from that child. There are many viruses of this type but they are all apparently host-specific; that is to say you cannot infect a mouse with a human strain and you cannot infect a man with a mouse strain or a hamster strain. There seem to be as many strains as there are small mammals—or large mammals too.

It is difficult to grow this virus. The cytopathic changes do not appear in the cell cultures until incubation has been prolonged for quite a few weeks—for much longer than we usually keep cultures going so that the type of culture ordinarily used is not very suitable.

I think one of the reasons why the affected cells are hard to find in the urine is that the cells tend to degenerate and disintegrate in a short time and the abnormal appearances are no longer recognizable. If we could devise some method of examining really fresh material we might see them more often.

*Prof. Neale:* Would there be any method at all of finding out in the ante-natal period whether the mother was carrying the infection?

*Dr. Cayton:* In theory this could be done by looking for antibodies but the antigen is not generally available.

*Dr. Lloyd:* But the virus can be grown?

*Dr. Cayton:* It can be grown but this is rather difficult.

*Dr. Corner:* If we saw another baby we suspected of having this infection should we send you a sample of its blood?

*Dr. Cayton:* It would be worth trying with blood and also urine. I think we now have cells in which the virus might grow.

*Question:* Are typical cells found in the saliva? It would seem easier to look for them there than in the urine.

*Dr. Cayton:* The virus is found in the saliva. I cannot say about the cells.

*Dr. Lloyd:* I should not think it would be easier to find them there than in the urine. It seems to me from what Dr. Brown has just shown us that what is needed is some sort of satisfactory biopsy of some organ such as salivary gland. This would seem to be likely to be much more reliable.

I am interested in your comparison with herpes simplex because the inclusion bodies look very much like herpes inclusions except that they are bigger and more obvious.

*Prof. Neale:* Has Dr. Brown any explanation of the thrombocytopenia?

*Dr. Brown:* No.

*Dr. Lloyd:* Were the megakaryocytes in the bone marrow normal?

*Dr. Brown:* They appeared to be. I think Professor Neale's own explanation may very well be right—that the thrombocytopenia was secondary.

*Dr. Lloyd:* Secondary to what?

*Dr. Corner:* That the purpura was due to liver damage and that the platelets were "used up" in the haemorrhagic areas.

*Question:* Why are the lymph nodes not affected?

*Dr. Brown:* I suppose it is because lymphoid tissue is not favourable for virus growth. We examined a number of lymph nodes and no changes were present.

*Dr. Lloyd:* I am surprised that the epidermis is spared. It is affected in herpes and other pox-like diseases.

*Prof. Neale:* By a process of evolution viruses become specifically related to a particular cell.

*Dr. Lloyd:* But this one seems to be fairly catholic in its choice of cell. This is the first case I have ever seen.

*Dr. Corner:* The first case we have ever seen here in Bristol too!

## Figures and Tables

**PLATE XVII f1:**
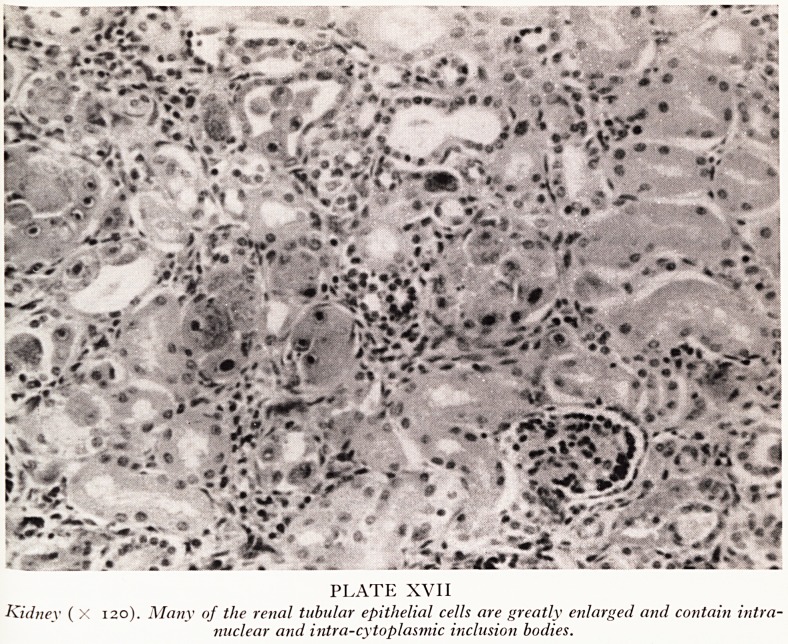


**PLATE XVIII f2:**